# The mediating role of self-care self-efficacy in the relationship between health behavior and psychological wellbeing among community-dwelling older adults with hypertension

**DOI:** 10.3389/fpubh.2025.1697089

**Published:** 2025-11-25

**Authors:** Shaimaa Mohamed Amin, Soher Ahmed Awad Abdel Aziz, Mohamed Hussein Ramadan Atta, Ahmed Farghaly Tawfik, Sally Mohammed Farghaly Abdelaliem, Nesreen AbdelMonaem AbouZeid, Mahmoud Abdelwahab Khedr, Ayman Mohamed El-Ashry

**Affiliations:** 1 Department of Psychiatric and Mental Health, and Community Health, College of Nursing, Qassim University, Buraydah, Saudi Arabia; 2 Gerontological Nursing, Faculty of Nursing, Assiut University, Asyut, Egypt; 3 Faculty of Nursing, Zarqa University, Zarqa, Jordan; 4 Psychiatric and Mental Health Nursing, Alexandria University, Alexandria, Egypt; 5 Nursing Department, College of Applied Medical Sciences, Prince Sattam Bin Abdulaziz University, Wadi Addawasir, Saudi Arabia; 6 Nursing Administration, Faculty of Nursing, Beni-suef University, Beni-suef, Egypt; 7 Department of Nursing Management and Education, College of Nursing, Princess Nourah bint Abdulrahman University, Riyadh, Saudi Arabia; 8 Department of Medical and Surgical Nursing, College of Nursing, Princess Nourah bint Abdulrahman University, Riyadh, Saudi Arabia; 9 Psychiatric and Mental Health Nursing, Faculty of Nursing, Alexandria University, Alexandria, Egypt; 10 Psychiatric and Mental Health Nursing, Department of Nursing, College of Applied Medical Sciences, Jouf University, Al Qurayyat, Saudi Arabia

**Keywords:** hypertension, health behavior, self-care self-efficacy, psychological wellbeing, geriatrics

## Abstract

**Background:**

Hypertension in older adults is a growing public health issue, often associated with decreased quality of life and increased healthcare demands. Health behaviors, self-efficacy in managing chronic illness, and psychological wellbeing are critical factors in effective hypertension control.

**Objective:**

To examine the mediating role of self-care self-efficacy in the relationship between health behaviors and psychological wellbeing among community-dwelling older adults with hypertension.

**Methods:**

A cross-sectional design was used, including 250 older adults attending outpatient geriatric clinics. Data were collected using a demographic questionnaire, the Arabic Geriatrics Health Behavior Questionnaire, the Arabic Self-Care Self-Efficacy Scale (SCSES), and the Psychological Wellbeing Scale.

**Results:**

Geriatric health behavior correlated positively with self-care self-efficacy and psychological wellbeing. Self-care self-efficacy was strongly associated with wellbeing. Self-care self-efficacy demonstrated a statistically significant partial mediating role between health behaviors and psychological wellbeing; the indirect effect (*β* = 0.399, *p* < 0.001) exceeded the direct effect (*β* = 0.140, *p* = 0.034). Education level showed a positive correlation with wellbeing, while age and longer hypertension duration were negatively correlated. The model explained 28.7% of the variance in self-care self-efficacy and 23.9% in wellbeing.

**Conclusions and recommendations:**

Self-care self-efficacy plays a partial mediating role between health behaviors and psychological wellbeing in older adults with hypertension. Interventions should enhance self-care self-efficacy and health education while tailoring support to those with lower literacy or longer disease duration. Promoting confidence in self-management may improve wellbeing, treatment adherence, and support healthier aging.

## Introduction

Hypertension is highly prevalent among older adults and significantly affects health outcomes. In Egypt, it represents a major public health concern, with an estimated prevalence of ~50% among adults aged ≥60 years (1) and ~28% in the general adult population (2). Care gaps are substantial, with awareness 37.5–43.9%, diagnosis 42.0–64.7%, treatment 24.0–54.1%, and medication adherence ~51.9% (2). These figures underscore the urgency of strengthening awareness efforts, consistent screening and diagnosis, and adherence-support programs to reduce cardiovascular risk and improve population health in Egypt.

These statistics underscore the urgent need for improved hypertension management strategies in Egypt. This includes implementing better awareness campaigns, maintaining consistent screening and diagnosis practices, and enhancing treatment adherence programs. Addressing these gaps is crucial for reducing the burden of cardiovascular diseases and improving the overall health outcomes for the Egyptian population.

In later life, hypertension contributes to poorer mental health through intertwined biological and psychosocial pathways. Chronically elevated blood pressure accelerates cerebral small-vessel disease with white-matter hyperintensities and reduced cerebral perfusion—features repeatedly linked to late-life depression, apathy, and cognitive slowing (3). Hypertension-related allostatic load also interacts with low-grade systemic inflammation and stress-axis dysregulation (HPA-axis), both of which are associated with depressive and anxiety symptoms in older adults (4). Beyond biology, treatment complexity and polypharmacy increase day-to-day burden and are themselves associated with non-adherence, adverse effects, and higher depressive symptomatology in hypertensive patients (5). Social sequelae—fear of complications, functional limitations, and reduced social engagement—can compound distress. Together, these mechanisms help explain the high co-occurrence of hypertension with depressive and anxiety symptoms in older populations and underscore the need to address mental health within hypertension care (6).

The relationship between health behaviors and psychological wellbeing among the older adult with hypertension is complex. Key health behaviors, including medication adherence, physical activity, and healthy dietary choices, are crucial for effective hypertension management and the prevention of related complications (7). However, these behaviors are significantly shaped by psychological factors like stress, anxiety, and depression, which are common among older adults dealing with chronic illnesses (8). Poor psychological wellbeing often leads to unhealthy behaviors, forming a challenging cycle where negative emotions reduce motivation for self-care, ultimately worsening hypertension and increasing health risks (9). Thus, addressing psychological wellbeing alongside promoting healthy behaviors is crucial to improving health outcomes in this population. Comprehensive interventions that consider both psychological and behavioral aspects can help break this cycle, fostering both better health behaviors and wellbeing in older adults with hypertension.

A vital component of the self-care dynamic is self-efficacy, defined as the belief in one’s ability to carry out specific health-related actions (10). This belief is a key factor in health management, as individuals with high levels of self-efficacy are more likely to adopt and sustain health-promoting behaviors, leading to improved health outcomes (11). For example, older persons with strong self-efficacy are more likely to effectively manage hypertension by adhering to their treatment plans and making healthy lifestyle choices (12). However, it is important to note that low self-efficacy can lead to poor treatment plan adherence and unhealthy lifestyle choices, which can have a detrimental effect on one’s physical and mental health (13).

Research has shown that self-efficacy significantly influences psychological wellbeing among hypertensive older adults. Higher levels of self-efficacy are associated with better mental health outcomes, including reduced levels of stress, anxiety, and depression. For instance, a study found that older adults with higher self-efficacy reported higher life satisfaction levels and lower levels of harmful effects (14). This relationship suggests that self-efficacy can serve as a protective factor, helping individuals manage the psychological challenges associated with hypertension. Enhancing self-care self-efficacy through targeted interventions may improve the psychological wellbeing and overall quality of life for this population (12). For example, a study found that self-efficacy significantly predicted adherence to self-care behaviors in hypertensive patients, associated with better psychological outcomes (15). This suggests that interventions to enhance self-care self-efficacy could effectively improve both health behaviors among older adults with hypertension (16).

### Significance of the study

Bandura’s Social Cognitive Theory, which emphasizes the importance of self-care self-efficacy in influencing behavior and mental health, provides a strong rationale for this study. According to Bandura (17), self-efficacy is the conviction that one can carry out the necessary steps to accomplish goals. People’s decisions, the amount of effort they put into their acts, their resolve in the face of adversity, and their capacity to overcome setbacks are all influenced by this idea. When managing hypertension in older adults, self-efficacy is crucial for health-related behaviors such as taking medications as prescribed, exercising, and eating a balanced diet—all of which are essential for controlling blood pressure and preventing complications (17).

Guided by Social Cognitive Theory, we conceptualize self-care self-efficacy as a proximal determinant of geriatric health behaviors (e.g., medication adherence, diet, physical activity). In SCT, self-efficacy works in concert with goals and outcome expectations to regulate action, motivation, and wellbeing (18, 19). In hypertension, higher self-efficacy is consistently linked to better self-care (12) and adherence across multiple behaviors (20). At the same time, SCT’s reciprocal determinism recognizes that successful performance of health behaviors can build self-efficacy via mastery experiences, especially in older adults engaged in day-to-day self-management (17). Accordingly, we prioritize the pathway self-efficacy → health behaviors → psychological wellbeing, while acknowledging that behavior may also reinforce efficacy over time. This framing clarifies our theoretical rationale and aligns our hypotheses with SCT applications in chronic-disease self-management. Understanding this path analysis may inform the development of interventions that enhance self-efficacy, ultimately leading to better health and psychological outcomes in this population.

### Aim

To examine the relationship between health behaviors, self-care self-efficacy, and psychological wellbeing among hypertensive older adults attending outpatient geriatric clinics at Assiut University Hospital.

### Research hypotheses

1. A positive association exists between health behaviors and psychological wellbeing in older adults with hypertension.
2. Self-care self-efficacy is positively associated with psychological wellbeing in older adults with hypertension.
3. Self-care self-efficacy may mediate the relationship between health behaviors and psychological wellbeing in older adults with hypertension.

## Subjects and methods

### Study design

A cross-sectional descriptive research design was utilized for this study, adhering to the guidelines established by the Strengthening the Reporting of Observational Studies in Epidemiology (STROBE) checklist. This approach ensures that the study’s reporting is both rigorous and transparent.

### Setting

The study was conducted at the outpatient geriatric clinics of Assiut University Hospital, which play a crucial role in providing specialized care to the older adult population in the region. These clinics are specifically designed to address the unique health needs of older adults, providing services such as chronic disease management, preventive care, and mental health support. Patients at these clinics benefit from a multidisciplinary team that includes geriatricians, nurses, physical therapists, and social workers, all working collaboratively to deliver comprehensive care. The primary focus is on maintaining the overall health and wellbeing of older adult patients, helping them effectively manage conditions such as hypertension, diabetes, and arthritis while also providing essential support for cognitive and emotional health.

### Study participants and sample size

The target group for this study was hypertensive older adults. Eligible participants were community-dwelling older adults aged 60 years and above, diagnosed with hypertension, and capable of performing daily self-care activities. Additionally, participants needed to be willing to participate in the study. Exclusion criteria included older adults residing in institutionalized settings, such as nursing homes or long-term care facilities, as the study focused on those living independently within the community. Furthermore, individuals with severe cognitive impairments, diagnosed mental health disorders that could interfere with the completion of study assessments, or acute medical conditions requiring immediate hospitalization were also excluded. These criteria were established to ensure a homogeneous study population capable of providing a reliable and valid response.

*A priori* power analysis was performed in G*Power 3.1.9.4 for multiple linear regression (fixed model, *R*^2^ deviation from zero) with effect size *f*^2^ = 0.15 (moderate), *α* = 0.05, power (1 − *β*) = 0.95, and 22 predictors (13 sociodemographic + 9 scale dimensions). The required minimum sample was *N* = 230. We over-recruited to *N* = 260 to allow for non-response; 250 complete cases were analyzed. However, six participants refused to participate, and four were ineligible, yielding an impressive response rate of 96.1%.

### Measurements of interest

#### Demographic form

The form encompasses a comprehensive range of elements, including sociodemographic details such as age, gender, education level, marital status, residence, family structure, employment status, and income. It further delves into medical history, family history of hypertension, and the duration since the diagnosis of hypertension.

#### The geriatrics health behavior questionnaire (GHBQ)

The questionnaire was developed by Bakhshandeh et al. (21) to assess health behaviors in older adults and translated into Arabic by Shaban et al. (22). It consisted of 17 items across seven subscales: physical activity (1 item with two parts), nutrition status (2 items), medication adherence (4 items), stress management (4 items), smoking and alcohol consumption (2 items), sleep quality (2 items), and checkups (2 items). Geriatrics Health Behavior Questionnaire (GHBQ) items are scored between 0 and 1 based on the accumulation approach. Total questionnaire scores range from 0 to 17, with higher values indicating better health behaviors. The Arabic version of GHBQ demonstrated excellent content validity with a Content Validity Index (CVI) average of 0.91. Reliability analysis revealed strong internal consistency, with Cronbach’s alpha values ranging from 0.74 to 0.87 and high test–retest reliability, as indicated by Spearman’s correlations ranging from 0.75 to 0.88. Confirmatory Factor Analysis (CFA) supported the factor structure with good fit indices, and criterion validity was confirmed through significant correlations with established health behavior measures.

#### Spanish version of the self-care self-efficacy scale (SCSES)

The original SCSES, developed by Yu et al. (23), consists of 10 items that measure “self-efficacy in self-care” on a single dimension. The Spanish version validated by Chica et al. (24) is also composed of 10 items but across four dimensions: Self-efficacy in self-care based on clinical knowledge (3 items), Self-efficacy in Self-Care-Maintenance (2 items), Self-efficacy in Self-Care-Monitoring (3 items), and Self-efficacy in Self-Care-Management (2 items). Each item is rated on a five-point Likert scale, ranging from 1 (not confident) to 5 (very confident), with higher scores indicating greater self-care self-efficacy in managing chronic conditions. The Spanish version of the SCSES exhibited excellent psychometric properties. It demonstrated strong construct validity with good fit indices from confirmatory factor analysis [Comparative Fit Index (CFI) = 0.970, Tucker–Lewis Index (TLI) = 0.953, Root Mean Square Error of Approximation (RMSEA = 0.080)]. The scale also showed acceptable internal consistency (Cronbach’s alpha > 0.7), with an overall alpha of 0.89, indicating strong reliability. After translation into Arabic, exploratory factor analysis revealed factor loadings ranging from 0.442 to 0.829 before rotation and from 0.580 to 0.953 after varimax rotation, all of which exceeded the 0.35 threshold and collectively explained 68.606% of the total variance.

#### Psychological wellbeing scale (PWBS)

The eight-item Psychological Wellbeing Scale [PWBS; (25, 26)] assesses psychological wellbeing using items rated on a 6-point Likert scale, ranging from 1 (strongly disagree) to 6 (strongly agree), where higher scores indicate better wellbeing. The scale demonstrated excellent internal consistency, with a Cronbach’s alpha of 0.91 and robust test–retest reliability, as evidenced by correlations of 0.73 over 1 month and 0.64 over 2 months. Construct validity was confirmed through exploratory factor analysis (EFA), which supported a single-factor structure, and CFA, which yielded good fit indices (CFI = 0.97, TLI = 0.95, RMSEA = 0.04). In this study, the scale’s reliability was strong with a Cronbach’s alpha of 0.88. After translating the scale into Arabic, exploratory factor analysis affirmed its content validity with satisfactory factor loadings both before and after varimax rotation, explaining 78.151% of the total variance. The Kaiser-Meyer-Olkin measure and Bartlett’s test also confirmed the data’s appropriateness for factor analysis, validating the scale for Arabic-speaking populations.

### Study procedures

#### Tool preparation and pilot study

The research instruments, including SCSES and PWBS, were translated into Arabic with meticulous care. Bilingual experts fluent in both English and Arabic undertook the translation to ensure both accuracy and cultural appropriateness. To validate the translations, a back-translation into English was performed to confirm linguistic equivalence and address any discrepancies that may have arisen. Following this, face validity assessments were carried out for each instrument. Expert panels reviewed the translated tools to ensure they effectively captured the intended constructs in the Arabic context.

Additionally, feedback from potential participants was solicited to verify the clarity, relevance, and cultural suitability of the translated items. Reliability was assessed using statistical methods, including Cronbach’s alpha, to ensure internal consistency. A pilot study involving 30 older adults was conducted to test the clarity, relevance, and reliability of the instruments. Participants from this pilot study were excluded from the main research. The pilot study results indicated that no modifications were needed, confirming the instruments’ suitability for the main study.

### Data collection

The data collection process began with a comprehensive orientation session for participants, during which the study’s objectives were clearly explained, and the voluntary nature of participation was emphasized. Researchers addressed all participants’ questions and concerns, highlighting the confidentiality measures in place to build trust. Data were collected by trained researchers from August to October 2024. The questionnaires were distributed in clinic waiting areas, allowing participants to complete them at their own pace. Data collection was conducted from Saturday to Thursday, between 10:00 a.m. and 2:00 p.m., to accommodate participants’ schedules and maximize response rates.

### Ethical considerations

Approval for this study was secured from the Research Ethics Committee of the Faculty of Nursing at Assuit University, Egypt, under reference number (1120230872). Additionally, permission was obtained from the clinic directors after a detailed explanation of the study’s objectives was provided. The research adhered to the ethical guidelines outlined in the Declaration of Helsinki, ensuring the protection of participants’ rights and wellbeing. Written informed consent was obtained from all participants after a comprehensive explanation of the study’s goals. Participants’ privacy and anonymity were strictly maintained, and all collected data were handled with the highest level of confidentiality. Participants were also informed of their right to withdraw from the study at any time without any consequences.

### Statistical design

Before being entered into the computer, the data was verified. The Statistical Package for the Social Sciences (SPSS version 25.0), created by IBM in Illinois, Chicago, USA, was used for this purpose, followed by data analysis and tabulation. Descriptive statistics were employed to present the characteristics of both study participants and variables. The mean scores were computed for numerical values. When *p* ≤ 0.00 L, a highly significant level value was considered, and the significance level was selected at *p* < 0.05. Parametric tests were employed, as the data demonstrated a normal distribution according to the Kolmogorov–Smirnov test. Variables’ multicollinearity was assessed, confirmed tolerance was >0.1, and the variance inflation factor (VIF) was <3, showing no multicollinearity. Pearson’s correlation was used to analyze bivariate correlations between the study variables. Spearman’s rank correlation was used to analyze bivariate correlations between the study variables and the ranked participants’ characteristics. To examine the mediating role of Self-care self-efficacy in the relationship between Geriatric health behavior and psychological wellbeing, a mediation analysis was conducted using Hayes’ PROCESS macro (Model 4) with 5,000 bootstrap samples to estimate the indirect effect. This approach allowed for robust testing of the mediation pathway, revealing that Self-care self-efficacy partially mediated the positive relation between Geriatric health behavior and psychological wellbeing (27, 28). For the power calculation we counted all potential covariates (13 sociodemographic) and scale dimensions (9) as candidate predictors, though the mediation model reported here focuses on total GHBQ, SCSES, and PWBS scores with sociodemographic covariates entered as controls.

## Results

Table 1 presents the demographic and clinical characteristics of study participants. The larger proportion of participants were male (61.2%) and aged 60–64 (34.8%), with approximately two-thirds married (67.6%) and more than half residing in urban areas (54.0%). Education levels varied, with 36.4% having basic education and 32.4% being illiterate. Nearly half of the participants reported insufficient income (52.0%). A significant proportion had a family history of hypertension (68.8%), and 36.8% had comorbid diabetes mellitus. The largest proportion had been diagnosed with hypertension for over 5 years (38.8%).

**Table 1:** Distribution of the study participants according to their characteristics (*n* = 250).

Variable	Category	Frequency	Percent
Age	60–	87	34.8
	65–	69	27.6
	70–	60	24.0
	75+	34	13.6
Gender	Male	153	61.2
	Female	97	38.8
Social status	Single	6	2.4
	Married	169	67.6
	Divorced	27	10.8
	Widow	48	19.2
Education level	Illiterate	81	32.4
	Basic	91	36.4
	Secondary	45	18.0
	University	33	13.2
Residence	Urban	135	54.0
	Rural	115	46.0
Job	Not working	66	26.4
	Self-employed	37	14.8
	Retired	77	30.8
	Manual work	30	12.0
	Farmer	40	16.0
Income	Enough	114	45.6
	Not enough	130	52.0
	Enough & save	6	2.4
Type of family	Nuclear	146	58.4
	Extended	104	41.6
Family history of HTN	Yes	172	68.8
	No	78	31.2
Comorbidities	No	103	41.2
	DM	92	36.8
	CKD	13	5.2
	Chronic heart disease	29	11.6
	Other	13	5.2
Duration since diagnosis with HTN	Less than 5	82	32.8
	5–	97	38.8
	10+	71	28.4
	Total	250	100.0

Table 2 presents descriptive statistics and bivariate correlations among the various study variables and their subdimensions. Geriatric health behavior is positively and significantly correlated with all its dimensions, especially stress management (*r* = 0.742) and medication adherence (*r* = 0.698), indicating these are key contributors. Self-care self-efficacy and its subdimensions (maintenance, monitoring, and management) exhibit strong intercorrelations (e.g., self-efficacy with SC maintenance: *r* = 0.901), suggesting a cohesive self-care construct. Psychological wellbeing is moderately correlated with most variables, particularly sleep quality (*r* = 0.417) and self-care management (*r* = 0.473), highlighting their importance for mental health. Negative correlations, such as between physical activity and smoking/alcohol (*r* = −0.156), and between nutrition and medication adherence (*r* = −0.147), suggest potential behavioral conflicts.

**Table 2:** Descriptive statistics and pairwise correlations of the study variables and their dimensions (*n* = 250).

	Study variables	1	2	3	4	5	6	7	8	9	10	11	12	13	14
1	Geriatric health behavior	1													
2	Physical activity	0.217**	1												
3	Nutrition status	0.268**	0.046	1											
4	Medication adherence	0.698**	0.112	−0.147*	1										
5	Stress management	0.742**	0.093	0.153*	0.367**	1									
6	Smoking & alcohol consumption	0.289**	−0.156*	0.017	0.079	0.119	1								
7	Sleep Quality	0.523**	0.220**	0.158*	0.239**	0.218**	0.085	1							
8	Checkup	0.182**	−0.155*	0.041	−0.109	−0.043	0.077	−0.073	1						
9	Self-care self-efficacy	0.535**	0.270**	0.059	0.443**	0.325**	0.162*	0.411**	−0.038	1					
10	Self-Care-Behaviors	0.497**	0.258**	0.088	0.384**	0.289**	0.161*	0.410**	−0.029	0.943**	1				
11	Self-Care-Maintenance	0.527**	0.239**	0.016	0.460**	0.328**	0.177**	0.365**	−0.026	0.901**	0.799**	1			
12	Self-Care-Monitoring	0.523**	0.260**	0.098	0.420**	0.335**	0.150*	0.366**	−0.035	0.932**	0.848**	0.814**	1		
13	Self-Care-Management	0.458**	0.245**	0.048	0.367**	0.297**	0.089	0.380**	−0.042	0.880**	0.835**	0.736**	0.773**	1	
14	Psychological wellbeing	0.354**	0.295**	0.166**	0.112	0.293**	−0.005	0.417**	−0.034	0.474**	0.429**	0.398**	0.433**	0.473**	1
Mean ± SD		8.39 ± 2.68	0.21 ± 0.33	0.53 ± 0.57	2.22 ± 1.36	2.21 ± 1.2	1.62 ± 0.45	1.03 ± 0.67	0.56 ± 0.73	33.51 ± 12.23	9.97 ± 3.95	6.65 ± 2.72	9.95 ± 3.88	6.88 ± 2.67	37.28 ± 9.06

*p < 0.05; **p < 0.01.

Table 3 presents correlations between participants’ characteristics and study variables: geriatric health behavior, self-care self-efficacy, and psychological wellbeing. The educational level shows significant positive correlations with all three variables, particularly with psychological wellbeing (*r* = 0.443**). Conversely, age is negatively correlated with psychological wellbeing (*r* = −0.219**). Income is negatively correlated with geriatric health behavior (*r* = −0.190**). Duration since hypertension diagnosis is negatively associated with psychological wellbeing (*r* = −0.204**), highlighting a potential decline in mental health over time.

**Table 3:** Correlations between study variables and study participants ranked characteristics (*n* = 250).

Participants’ characteristics	Geriatric health behavior	Self-care self-efficacy	Psychological wellbeing
Age	0.009	−0.074	−0.219**
Educational level	0.298**	0.228**	0.443**
Income	−0.190**	−0.082	−0.101
Duration since diagnosis with HTN	0.001	−0.029	−0.204**

*p < 0.05; **p < 0.01; statistical significance based on two-tailed correlation tests.

Table 4 reveals that geriatric health behavior significantly influences psychological wellbeing both directly and indirectly through self-care self-efficacy. Geriatric health behavior strongly predicts self-care self-efficacy, which in turn has a substantial positive effect on psychological wellbeing. The indirect effect through self-care self-efficacy is notably stronger than the direct effect, suggesting that self-care self-efficacy plays a key mediating role in enhancing psychological wellbeing among older adults. Model summaries show good explanatory power, with geriatric health behavior accounting for 28.7% of the variance in self-care self-efficacy and the combined model explaining 23.9% of the variance in psychological wellbeing.

**Table 4:** Mediation analysis of the effect of geriatric health behavior on psychological wellbeing via self-care self-efficacy.

Model	Outcome variable	Predictor/s	*β*	SE	*t*	*p*	95% CI	
							(LLCI)	(ULCI)
1	Self-care self-efficacy (Mediator)	Geriatric health behavior	0.535	0.245	9.985	0.000	1.964	2.929
2	Psychological wellbeing (Outcome)	Geriatric health behavior	0.140	0.2225	2.1297	0.0342	0.0356	0.9120
		Self-care self-efficacy	0.399	0.0487	6.0731	0.000	0.1998	0.3916	
	Direct Effect	Geriatric health behavior	0.140	0.2225	2.1297	0.0342	0.0356	0.9120	
	Indirect Effect via Geriatric Health Behavior	Geriatric health behavior	0.214	0.046			0.127	0.305

Model summary	
Model 1	*R* = 0.54, *R*^2^ = 0.287, *F* (1,248) = 99.70, *p* = 0.000, MSE = 107.14
Model 2	*R* = 0.488, *R*^2^ = 0.239, *F* (2,247) = 38.74, *p* < 0.000, MSE = 62.99

## Discussion

The national epidemiological context supports our findings. In Egypt, adult hypertension prevalence is ~28%, with documented gaps in awareness, diagnosis, and treatment, which can attenuate self-care and wellbeing (2). These gaps underscore the importance of interventions that build self-care self-efficacy and target multiple behaviors within routine services, as argued below.

The relationship between health behavior and psychological wellbeing among community-dwelling older adults with hypertension is significantly mediated by self-care self-efficacy. Positive health behaviors can enhance individuals’ confidence in managing their health effectively. The current study aimed to investigate the mediating role of self-care self-efficacy in the relationship between health behavior and psychological wellbeing among community-dwelling older adults with hypertension.

Among older adults dealing with hypertension, health behaviors influence psychological health via multiple linked pathways. For example, regular adherence to prescribed medications helps maintain steady blood pressure levels, which in turn eases the physical tension tied to symptoms such as persistent headaches or exhaustion, ultimately reducing anxiety and promoting more balanced emotional states (11). Engaging in regular exercise boosts the production of endorphins and supports brain plasticity, helping to offset the cognitive slowdowns that come with aging and instilling a greater sense of energy that protects against depression (29). Likewise, practices for managing stress—like mindfulness exercises or deep breathing—disrupt the ongoing overactivity of the hypothalamic–pituitary–adrenal (HPA) axis, a common issue in seniors with long-term illnesses, resulting in lower cortisol production and stronger emotional coping abilities (30). Beyond these bodily effects, such behaviors work through mental processes as well: consistently following healthy routines builds feelings of competence and autonomy, which boost self-worth and overall contentment while countering the sense of powerlessness often experienced in ongoing health challenges (17). In the long run, these habitual actions form reinforcing cycles, as better physical results (such as improved rest from fewer hypertension issues) ease mental strain even more, disrupting the pattern where emotional difficulties worsen lapses in self-care (7).

The study highlights complex relationships among geriatric health behavior, self-care self-efficacy, psychological wellbeing, and their respective components. It shows that geriatric health behavior is closely linked to stress management and medication adherence, indicating these areas are vital for overall health in older adults. This implies that interventions focused on these specific aspects could lead to significant improvements in health behaviors within this population (31). Moreover, self-care self-efficacy, which includes maintenance, monitoring, and management, displays strong intercorrelations, indicating that enhancements in one area may positively affect others.

Psychological wellbeing correlates significantly with sleep quality and self-care management, underscoring their importance for mental health among older adults. Research indicates that better sleep quality is associated with improved emotional health and a reduced risk of issues like depression and anxiety (32). Likewise, effective self-care management, which reflects an individual’s belief in their ability to handle chronic conditions, is linked to higher life satisfaction and lower stress levels (33). These findings highlight the necessity for comprehensive interventions that address both physical health and psychological wellbeing (34).

The data also uncovers potential behavioral conflicts, as seen in negative correlations between certain health behaviors. For example, the inverse relationship between physical activity and smoking/alcohol consumption suggests that those who are more physically active tend to smoke or drink less (35). Similarly, the negative correlation between nutrition and medication adherence may reflect difficulties in balancing healthy eating with consistent medication routines. These conflicting behaviors illustrate the complexities involved in changing health behaviors and the need for tailored interventions that consider everyone’s unique challenges and motivations (36).

The analysis reveals significant connections between participant characteristics and key study variables, providing insights into how socio-demographic factors intertwine with health behaviors, self-efficacy, and psychological wellbeing. Education level shows a positive correlation with all three variables, revealing that higher education may enable older adults to adopt healthier behaviors and enhance their confidence in managing their health, ultimately improving their mental state. Increased education can improve health literacy and access to resources that promote wellbeing (36, 37).

In contrast, age is negatively correlated with psychological wellbeing, indicating a decline in mental health as individuals grow older. Factors such as deteriorating physical health, social isolation, and increased life stressors can contribute to this decline (38). Additionally, income negatively correlates with geriatric health behavior, denoting that lower-income individuals may struggle to maintain healthy behaviors due to limited access to resources like nutritious food and safe spaces for physical activity (39).

Moreover, the duration since a hypertension diagnosis is negatively associated with psychological wellbeing, suggesting that living with a chronic condition can lead to a gradual decline in mental health. The ongoing management of hypertension and the associated stress, anxiety, and potential complications may adversely affect psychological wellbeing (40, 41). The detrimental effects of long-term hypertension on psychological health arise from ongoing processes, such as the “weathering” phenomenon, in which persistent inflammation due to poorly managed blood pressure gradually damages brain connections, increasing susceptibility to depressive disorders (40). With time, as the condition persists, frequent episodes of worsening symptoms or related issues deplete an individual’s emotional reserves, fostering a state of learned helplessness and reduced drive to engage in self-care practices, thereby sustaining a downward spiral in mental wellbeing (41).

Hypertension significantly influences the overall quality of life (QoL) in older patients, frequently undermining their day-to-day performance in physical, emotional, and social spheres. For older adults living independently in the community, when blood pressure remains poorly controlled, it intensifies issues such as tiredness and lightheadedness that restrict routine tasks and self-reliance; at the same time, it raises the chances of heart-related incidents, sparking greater anxiety and pulling people away from social interactions, which in turn weakens relationships and reduces personal fulfillment (13). The persistent strain of this condition often hampers QoL by creating feelings of reliance on others, especially in cases involving co-existing issues like diabetes—seen in 36.8% of participants in our study—that worsen movement difficulties and daily care demands, leading to poorer ratings in standard QoL assessments (such as SF-36 areas related to energy levels and emotional wellbeing) (42). That said, our results point to how adopting beneficial health habits and stronger self-care confidence can help offset these downsides, through improved condition management and mental toughness, fostering greater independence and less reliance on medical services in the long term (12).

The mediation analysis underscores the significant role of self-care self-efficacy in bridging geriatric health behavior and psychological wellbeing. Findings indicate that geriatric health behavior strongly influences self-care self-efficacy, which subsequently enhances psychological wellbeing. This reveals that interventions aimed at improving health behaviors in older adults could be more effective if they also focus on boosting self-care self-efficacy. Strengthening individuals’ confidence in their health management can lead to improvements in their psychological wellbeing (43).

The analysis shows that the indirect effect of geriatric health behavior on psychological wellbeing through self-care self-efficacy is stronger than the direct effect, emphasizing self-efficacy’s critical mediating role. This relationship indicates that the positive effects of health behaviors on mental wellbeing depend significantly on individuals’ confidence in executing those behaviors. This aligns with Bandura’s Social Cognitive Theory, which highlights self-efficacy’s importance in shaping behavior and mental health (44). Consequently, interventions should prioritize strategies to enhance self-efficacy, such as education, skills training, and opportunities for effective self-management (45).

In the context of this mediation model, the process unfolds in two key stages: first, adopting positive health practices fosters self-efficacy through hands-on successes (for example, sticking to a balanced diet and seeing blood pressure drop can solidify confidence in one’s abilities), paving the way for mental health gains via better control over emotions and less worry about worsening conditions (17). For older adults, this route holds special power since building self-efficacy helps counteract the pressures of age-related stereotypes, where feelings of weakness can sap emotional strength, enabling ongoing healthy habits to offer indirect protection for overall mental health (12).

Model summaries further support these conclusions, indicating that geriatric health behavior accounts for a substantial portion of the variance in self-care self-efficacy, while the combined model explains a significant amount of variance in psychological wellbeing. This illustrates that interventions targeting both health behaviors and self-care self-efficacy can lead to meaningful improvements in the psychological wellbeing of older adults. These findings reinforce the importance of a holistic approach to geriatric care, addressing both physical and psychological dimensions to promote healthy aging and overall wellbeing.

## Strengths and limitations

This study has several strengths that enhance the reliability and applicability of its findings. First, it employs a cross-sectional descriptive research design, adhering to the STROBE guidelines to ensure rigorous and transparent reporting. The use of well-validated and culturally adapted tools, including the Arabic versions of the Geriatric Health Behavior Questionnaire (GHBQ), Self-care Self-Efficacy Scale (SCSES), and Psychological Wellbeing Scale (PWBS), adds robustness to the data by ensuring that assessments are both reliable and contextually appropriate. Additionally, the use of G*Power software to determine an adequate sample size with high statistical power (95%) further strengthens the validity of the study’s conclusions. The study’s high response rate (96.1%) enhances its generalizability, as it reflects strong participant engagement and minimizes the potential for selection bias.

However, the study has limitations. Being cross-sectional in design, it captures a snapshot of associations but cannot establish causation between health behaviors, self-care self-efficacy, and psychological wellbeing. The focus on community-dwelling older adults with hypertension, excluding those in institutionalized care, limits the generalizability to the broader geriatric population. Additionally, self-reported data may introduce response bias, as participants might overestimate or underestimate their behaviors and self-care self-efficacy levels. While the cultural adaptation of the scales was undertaken meticulously, nuances may still exist in how certain concepts are perceived across different Arabic-speaking subcultures, which could affect the study’s applicability outside the specific demographic studied. Lastly, conducting the study within clinic hours and requiring participants to complete questionnaires in a public setting may have introduced environmental stress, which could have influenced their responses.

## Conclusion

This study demonstrated that self-care self-efficacy plays a partial mediating role in the relationship between health behaviors and psychological wellbeing among community-dwelling older adults with hypertension. Geriatric health behaviors such as stress management, medication adherence, and sleep quality were significantly associated with greater wellbeing, while higher education predicted better outcomes. Conversely, advancing age and longer duration of hypertension were linked to poorer psychological wellbeing. The findings highlight the importance of both behavioral and psychosocial factors in the management of hypertension in older adults.

### Implications for nursing practice

Nursing interventions should prioritize strategies that build confidence in self-care, such as tailored education, skills training, and motivational support, to improve both treatment adherence and psychological wellbeing. Stress management, medication adherence, and sleep quality emerged as strong predictors of wellbeing; therefore, nursing programs should integrate behavioral counseling and monitoring in these areas. Older adults with lower literacy or longer disease duration may need more structured guidance and support. Nurses should adapt communication methods and provide simplified, culturally appropriate educational resources. Considering the interplay of sociodemographic and clinical factors, nursing practice should adopt a comprehensive approach that addresses not only physical health but also psychological and social dimensions to enhance quality of life in hypertensive elders.

Antihypertensive therapy (AHT) guideline-directed pharmacologic blood-pressure control supported by home BP monitoring—works best when older adults have high self-care self-efficacy, i.e., confidence to organize and execute daily health tasks. In Social Cognitive Theory, self-efficacy regulates action, persistence, and self-management, and is strengthened through mastery, modeling, and affect regulation (17–19). In late-life hypertension, higher self-efficacy is consistently associated with better self-care and adherence across behaviors (12). In our sample, the health behaviors most tightly linked to wellbeing were stress management, medication adherence, and sleep quality, underscoring the value of brief nurse-led strategies that build mastery (teach-back + 7-day pillbox), vicarious learning (peer demonstrations of pill-taking and BP checks), persuasive planning (short goal-setting with if-then plans), and affect regulation (2-min paced breathing before BP checks) (12, 17–19). These are aligned with the study’s finding that targeting both health behaviors and self-care self-efficacy can meaningfully improve psychological wellbeing in older adults with hypertension.

For older adults living with a co-resident, convert support into simple dyadic routines: a weekly “pillbox Sunday” filled together; a shared alternate-day BP calendar; autonomy-supportive prompts for short walks; and quick side-effect checks that trigger timely clinician contact—leveraging efficacy pathways from mastery, modeling, and supportive persuasion (18, 19). For those living alone, lean on automation and community: alarms tied to daily anchors, large-font pictorial medication charts or pharmacy blister packs, brief remote check-ins (phone/WhatsApp) twice weekly, and a 4–6-person peer group that posts BP readings or steps—reducing cognitive load while sustaining adherence and wellbeing.

Taken together, these AHT-focused, efficacy-building strategies translate the mediation pattern observed in our data into practical routines that can be deployed in routine geriatric care and community settings, with minor tailoring by living situation.

## Data Availability

The raw data supporting the conclusions of this article will be made available by the authors without undue reservation.
